# Single-cell immune landscape of cerebrospinal fluid in non-HIV cryptococcal meningitis

**DOI:** 10.1186/s12916-026-05142-x

**Published:** 2026-08-10

**Authors:** Zai-Jie Jiang, Yuan-Yuan Wang, Yu-Ying Pan, Shi-Ying Zhang, Bi-Wei Lin, Jun-Mei Wang, Jian-Chen Hong, Xue-Wen Cheng, Chang-Bin Chen, Xiang-Ping Yao

**Affiliations:** 1 Department of Neurology, Fujian Institute of Neurology, the First Affiliated Hospital, Fujian Medical University, Fuzhou, 350005 China; 2 Joint Laboratory for Biomedical Research and Pharmaceutical Innovation, The Unit of Pathogenic Fungal Infection & Host Immunity, Shanghai Institute of Materia Medica, Chinese Academy of Sciences, Shanghai, China; 3 Department of Colorectal Surgery, The First Affiliated Hospital, Fujian Medical University, Fuzhou, 350005 China; 4 Lin Gang Laboratory, Shanghai, 201602 China; 5 Department of Rare Diseases, National Regional Medical Center, Binhai Campus of the First Affiliated Hospital, Fujian Medical University, Fuzhou, 350212 China

**Keywords:** Cryptococcal meningitis, Single-cell RNA sequencing, Cerebrospinal fluid, T cell receptor sequences, The CCL3/4-CCR5 signaling

## Abstract

**Background:**

Cryptococcal meningitis (CM), caused by *Cryptococcus *spp., ranks as the most severe fungal infection, accounting for approximately 200,000 deaths globally each year. However, the intricate interplay between disease severity and the host immune response remains incompletely understood, especially the nuanced immune responses driving CM progression.

**Methods:**

We conducted single-cell RNA sequencing paired with assembled T cell receptor sequencing on human cerebrospinal fluid (CSF) samples, including those from five patients with paired untreated and treated samples, alongside eight controls from patients with non-CNS inflammatory diseases (NCID). All participants were HIV-negative.

**Results:**

We unexpectedly observed an expansion of highly metabolically activated Macrophages_MRC1 in the CSF of untreated CM (UCM) patients, characterized by enriched apoptosis and M2-polarization signatures, as well as an expansion of exhausted CD4^+^ Temra_KLRB1 cells. As expected, this phenomenon was alleviated in the CSF of the treated CM (TCM) group. Moreover, repertoire analysis revealed a broader clonal T cell expansion in the UCM group compared with the NCID and TCM groups. Notably, C-C motif chemokine receptor 5 (CCR5) and its ligands CCL3 and CCL4 were upregulated in CD4^+^ Temra_KLRB1 cells in the CSF of CM patients. Importantly, CCL3 and CCL4 levels showed a positive correlation with CSF leukocyte counts, suggesting that CCL3/4-CCR5 signaling may represent a key mechanism underlying the *Cryptococcus*-specific immune response.

**Conclusions:**

Taken together, our findings describe the CSF immune compartment in CM patients and indicate that the antifungal response is compromised in this context.

**Supplementary Information:**

The online version contains supplementary material available at https://doi.org/10.1186/s12916-026-05142-x.

## Background

Cryptococcal meningitis (CM), caused by the invasion of *Cryptococcus* spp. into the central nervous system (CNS), is the most common form of fungal meningitis and represents the most devastating manifestation of cryptococcosis, accounting for approximately 200,000 deaths worldwide each year [1, 2]. Long-term severe neurological sequelae are prevalent in CM patients, including memory loss, visual impairment, hearing and language disorders, and motor deficits [3, 4]. Current therapeutic protocols include combinations of antifungal drugs, such as amphotericin B, flucytosine and fluconazole. However, due to poor tolerance of amphotericin B, even with antifungal treatment, the disease poses significant challenges, including low treatment efficacy, prolonged treatment duration, and substantial side effects. Notably, the mortality rate remains 24.8% even with recommended antifungal regimens [5]. Given the high mortality and limitations of existing treatments, there is an urgent need to better understand the host immune response to *Cryptococcus* to inform the development of new drugs and therapeutic strategies. Additionally, studies have demonstrated that an excessive anti-*Cryptococcus* inflammatory response is associated with disabling outcomes and mortality in CM patients. Thus, clarifying immune system alterations in CM patients is critical for developing targeted therapies and improving patient outcomes. However, the antifungal immune response in the host’s CNS following cryptococcal infection remains largely undetermined [6–8].

Host immune responses against *Cryptococcus* within the CNS can be broadly categorized into innate and adaptive immunity, both of which play critical roles in controlling *Cryptococcus* infection. Macrophages, neutrophils, and dendritic cells recognize *Cryptococcus*-related molecular patterns, activate relevant signaling pathways, and initiate immune responses [9–11]. Macrophages act as primary responders, clearing *Cryptococcus* through phagocytosis. Subsequently, these cells, along with other antigen presenting cells (APCs), assume an antigen-presenting role, bridging innate and adaptive immunity by stimulating T cells [12, 13]. CD4^+^ T helper cells secrete cytokines that activate and guide macrophages, cytotoxic T cells, and other effector cells to target the fungal pathogen [13]. In addition, B cells produce antibodies that neutralize fungal antigens and activate the complement cascade, leading to fungal lysis [14]. However, when host immune function is impaired or compromised, *Cryptococcus* can evade host immune surveillance by thickening its cell wall, rendering it resistant to immune attack [15–17]. Furthermore, the fungus can alter the differentiation environment and protein expression of phagocytic cells, promoting the polarization of M2 macrophages, a process that facilitates *Cryptococcus* survival and proliferation [18–20]. Recent studies indicate that CSF provides molecular signals that modulate the composition of myeloid cell populations within the CSF [21]. Yet, it remains unclear whether alterations in the CSF immune landscape are associated with disease severity, particularly when comparing treated versus untreated CM.

Currently, most research on *Cryptococcus*-induced immune responses in the CNS lies on murine models, with a lack of validation using clinical human samples. Moreover, studies using clinical specimens such as CSF have been limited to cytokine and chemokine profiling via Luminex or ELISA [3, 22]. These bulk analysis approaches measure the average transcriptional signal across millions of cells, thereby obscuring differences between individual cells. Given these limitations and the inherent diversity of immune cells, conducting single-cell sequencing for CSF immune profiling using samples from CM patients is an urgent research priority.

In this study, we collected 11 CSF samples, including those from five CM patients (both before and after treatment) and samples from non-CM patients. We then performed single-cell RNA sequencing (scRNA-Seq) and single-cell T cell receptor sequencing (scTCR-seq). Our results delineate the CSF immune landscape in CM patients and characterize transcriptional changes in immune cells across different disease stages. We also identified associations between disease stage and the host immune response, providing valuable resources analyzing the inflammatory features of CM patients. In summary, our data and findings may enhance understanding of CM pathogenesis and offer insights to inform the development of targeted therapies for the disease.

## Methods

### Patient cohort and clinical characteristics

Five patients with CM were admitted to the First Affiliated Hospital of Fujian Medical University and enrolled in the study from August 2022 to May 2023. All the five patients were diagnosed with definite CM, meeting the diagnostic criterion, at least one of the testing methods was positive in the CSF samples, including ink staining, fungal culture, metagenomic next-generation sequence (mNGS), and cryptococcal antigen (CrAg) test. Additionally, eight patients who had a CSF leukocyte count < 5 × 10^6^/L, and were excluded from CNS infection were included as the non-CNS inflammatory diseases group which included two cases of non-purulent cerebral venous and sinus thrombosis (CVST), two of epileptic attack, one of bilateral venous sinus stenosis (VSS), one of narcolepsy and cataplexy, one of transient ischemic attack (TIA), and one of benign intracranial hypertension syndrome. All study participants, including both CM patients and NCID controls, were HIV-negative. Lumbar puncture was performed within 48 h after admission, and 5–10 mL CSF samples were collected. And for CM patients, CSF samples were also collected approximately one month after treatment as TCM group. Detailed clinical characteristics, including gender, age, clinical symptoms, CSF examinations, and imaging findings, were collected for all groups. This study was approved by the ethics committee of the hospital and written informed consents were obtained from each patient or their guardian.

### CSF sample preparation for sequencing

CSF samples were centrifuged at 500 g at 4℃ for 10 min to precipitate the CSF cells. The supernatant was carefully aspirated, and 2 mL of red blood cell (RBC) lysis buffer was added to the precipitated cells to remove the RBC from the puncture injury. At 15–25 °C, the cells were centrifuged at 500 g for 5 min. After the supernatant was abandoned, the cells were washed twice with phosphate-buffered saline (PBS), and finally, the cells were re-suspended with 1mL PBS. Take 10 µL of the re-suspension, and stain with Trypan blue, then detect the proportion of surviving cells under the microscope and calculate the cell concentration. The concentration of cell suspension was adjusted to 1 × 10^5^/mL using PBS.

### Library construction

Employing the Singleron Matrix^®^ platform, single-cell suspensions were deposited onto a microwell chip. Barcoding beads were retrieved from the microwell chip to synthesize cDNA through mRNA reverse transcription. The cDNA libraries were prepared through PCR amplification, enzymatic fragmentation, and adapter ligation using the GEXSCOPE^®^ Single Cell RNA Library Preparation System according to the manufacturer’s protocols (Singleron GEXSCOPE Single Cell RNA-seq Library Kit, Singleron Biotechnologies) [23]. Individual libraries were sequenced on Illumina NovaSeq 6000 with 150 bp paired end reads.

### Primary analysis of raw read data

Raw RNA data were processed using the CeleScope (https://github.com/singleron-RD/CeleScope) v1.9.0 pipeline to generate gene expression matrices. Low-quality reads were filtered out, and poly-A tails and adapter sequences were trimmed using Cutadapt (version 1.17) [24]. Cell barcodes and unique molecular identifiers (UMIs) were extracted, and reads were mapped to the reference genome GRCh38 (Ensembl version 92 annotation) using STAR (version 2.6.1a) [25]. UMI and gene counts for each cell were obtained using feature Counts software (version 2.0.1) [26], generating expression matrix files for subsequent analysis.

### Quality control, dimension-reduction and clustering

Scanpy (version1.8.2), a Python 3.7 package [27], was utilized for quality control, dimensionality reduction, and clustering of the scRNA-seq data. Each sample dataset underwent filtering based on the following criteria: cells with gene counts less than 200 or in the top 2%, cells with high UMI counts (top 2%), cells with mitochondrial content exceeding 15%, genes expressed in fewer than 5 cells. After quality control, 129,470 cells retained for downstream analyses, with an average of 963 genes and 2,256 UMIs per cell. To reduce the dimensionality of sc-RNA-seq dataset, principal component analysis (PCA) was applied to the scaled variable gene matrix, and the top 20 principal components were used for downstream analyses, as determined by the Seurat selecting the top 2000 variable genes. The Louvain algorithm with a resolution parameter of 1.2 was employed to separate cells into 20 clusters. The Uniform Manifold Approximation and Projection (UMAP) was used for visualizing the cell clusters. The batch effect was eliminated by Harmony v1.0 [28] with the top 20 main components from PCA.

### Cell type annotation and subtyping of major cell type

Cellular identities were determined through Cell-ID software which employs gene signatures and hypergeometric tests (HGT) for cell identity recognition [29]. Dimensionality reduction and gene ranking are used to identify featured gene sets for each cell. HGT were performed against a brain reference database from SynEcoSys database [29], and cell identities are determined based on HGT p-values. Cluster annotation is done by calculating the frequency of each cell type within each cluster and assigning the dominant cell type as the cluster’s identity. Additionally, three immune cell types were subset and further clustered into sub-clusters by using the following parameters ‘CD4^+^ Temra_KLRB1 = 0.5, Monocytes = 0.5, Macrophages = 0.5’.

### Differentially expressed genes (DEGs) analysis

DEGs were identified using the Seurat FindMarkers function with default parameters, selecting genes expressed in over 10% of cells in a cluster and with an average log (Fold Change) greater than 0.25, and displayed the expression of markers of each cell type with heatmaps, dot plots, and violin plots that were generated with Seurat DoHeatmap, DotPlot, and Vlnplot function. Doublet cells expressing markers for multiple cell types were manually removed.

### Pathway enrichment analysis

To illustrate the potential functions of DEGs, Gene Ontology (GO) and Kyoto Encyclopedia of Genes and Genomes (KEGG) analyses were performed using the “clusterProfiler” R package (version 3.16.1) [30]. Pathways with adjusted *p*-values < 0.05 were considered significantly enriched. Additionally, Results of the significantly differential pathways were visualized using bar plots.

### Pseudotime trajectory analysis

Cell differentiation trajectories were constructed using Monocle2 (version 1.99) [31], with highly variable genes guiding the sorting of cells in order of spatial temporal differentiation. The DDRTree algorithm was implemented for variable feature selection and dimensionality reduction. Next, CytoTRACE (version 0.3.3) [32] was employed to predict the differentiation potential of monocyte subpopulations.

### Cell-cell interaction analysis

Intercellular communication networks were analyzed using CellChat [33] (v0.0.2) on scRNA-seq data. A CellChat object was generated through the R package process, followed by integration of cellular metadata into the designated object slot. The ligand-receptor interaction database was configured, and receptor-ligand matching inference was computationally implemented.

### TCR analysis

TCR clonotypes were assigned using the CeleScope (version1.9.0) V(D)J pipeline with GRCh38 reference genome. Subsequently, TCR diversity metrics integrating clonotype frequency distributions and cellular barcode associations were quantified. T cells co-expressing functional T cell receptor α-chain (TRA) and β-chain (TRB) were retained for downstream analysis based on stringent V(D)J recombination criteria requiring single productive TRA and TRB transcripts. Each unique TRA(s)-TRB(s) pair was defined as a clonotype. Clonality was determined by the presence of the same clonotype in at least two cells, and the degree of clonality was calculated based on the number of cells sharing the same pair.

### Flow cytometry

The experimental group consisted of CSF samples collected from diagnosed non-HIV CM patients before treatment, while the control group was a mixture of CSF samples from three non-HIV patients with non-CNS inflammatory diseases. Detailed clinical characteristics of all participants were provided in Additional file 2: Table S1. The CSF cell apoptosis was detected with Annexin V-APC/7AAD Apoptosis Detection kit according to the manufacturer’s instructions (Suzhou 4 A Biotech Co., Ltd., #FXP147). Visualization was achieved on the FACS Canto II (BD Biosciences).

### Luminex assay

Cytokine measurements were performed on CSF samples obtained from 10 control patients and 28 patients with CM. All of these participants were HIV-negative, whose detailed clinical characteristics were also presented in Additional file 3: Table S2. The Bio-Plex Pro Human Cytokine Screening 48-plex Panel (#12007283; Bio-Rad Laboratories, Hercules, CA, USA) was used to analyze cytokine levels in the CSF samples according to the manufacturer’s instructions. Results were expressed in pg/mL, and values above or below the detection limit were assigned the highest or lowest value from the standard curve, respectively. For statistical analysis, concentrations below the detection limit were converted to the lowest detectable value.

### Statistical analysis

Continuous variables were presented as medians with interquartile ranges, while categorical variables were expressed as counts or percentages. Student’s t-test and Mann-Whitney U test were employed for comparing continuous variables with normal and non-normal distributions, respectively. The Spearman’s rank correlation test was performed to assess the associations between CSF cytokines and CSF parameters, including leukocytes, proteins, and the CSF/blood glucose ratio. Statistical significance was set at *p* < 0.05 (two-tailed). Data analysis was conducted using R (version 4.2.3) and GraphPad version 9 (GraphPad Software, San Diego, California, USA).

## Results

### A single-cell level cell atlas of CSF in CM patients

**Fig. 1:**
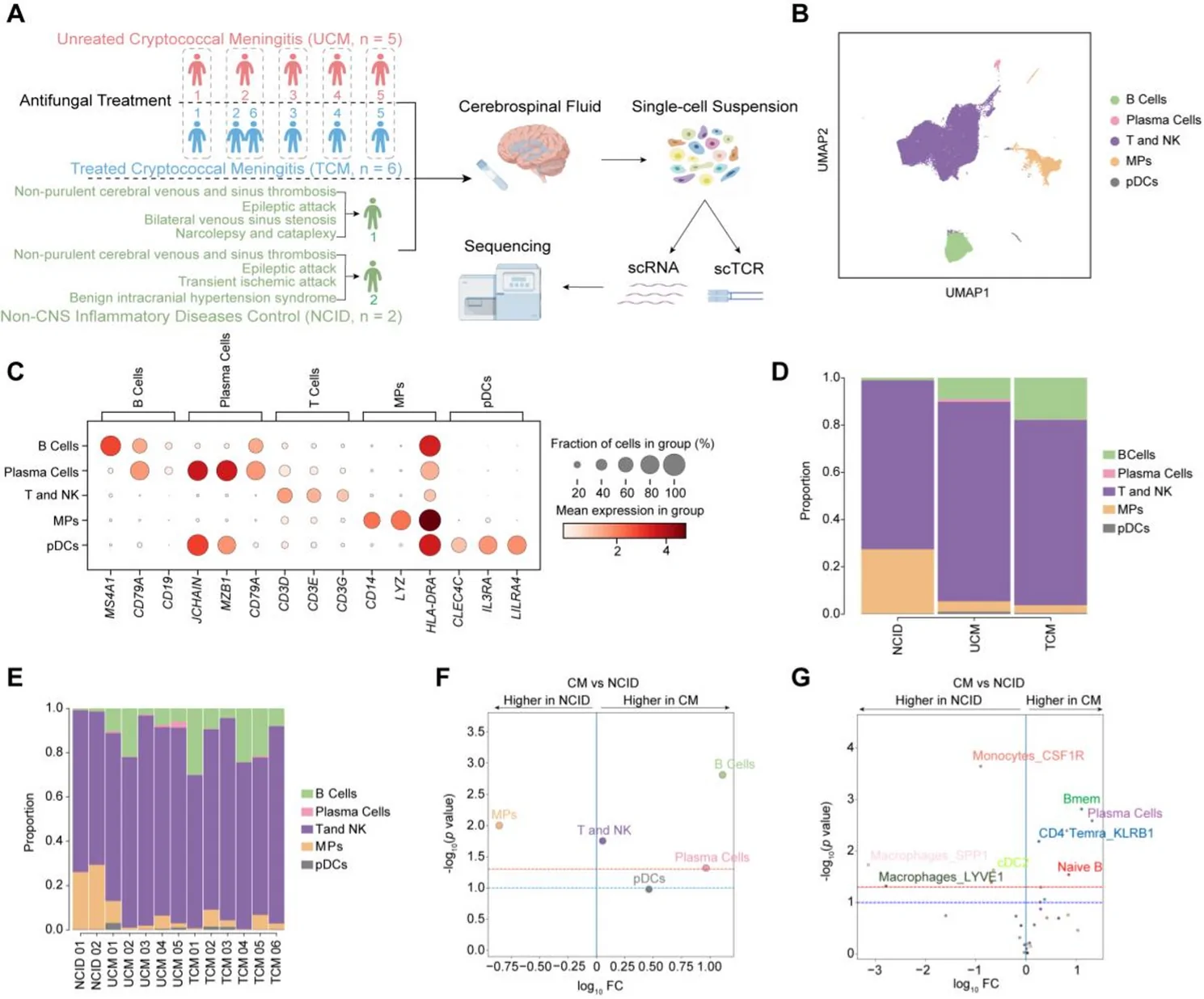
Immune landscape of CM CSF via scRNA-seq. **A** Schematic of the study design. CSF samples were obtained via lumbar puncture. Single cells were loaded into micro-wells and libraries were amplified for whole transcriptome or targeted TCR sequencing. **B** Uniform manifold approximation and projection (UMAP) plot showing clusters of CSF immune cells. **C** Bubble plot showing marker genes for each cell cluster. Color intensity denotes average expression. Dot sizes represent percentages of cells expressing the gene. **D** Stacked bar chart depicting relative proportions of cell types in different groups. **E** Compositions of cell types per sample. **F**-**G** Scatterplot depicting differences of cell type clusters (**F**) and cell subtype abundances (**G**) in ICM (n = 11) compared to NCID (n = 2). Horizontal line indicates the significance threshold (*p* = 0.05). *p*-values are determined by unpaired two-tailed Student’s *t*-test

To identify and characterize the CSF-infiltrating immune cells in CM patients, we collected 11 CSF samples from 5 CM patients for microfluidic microchip-based single-cell transcriptome sequencing. These CM samples were obtained at two distinct time points: before and after antifungal treatment. Samples collected prior to treatment were designated as the untreated CM group (UCM), and those collected post-treatment as the treated CM group (TCM). For joint analysis, these comprised the integrated CM (ICM). A one-month treatment course was administered between the collection of these two sets of samples. Following treatment, patients showed significant improvement in symptoms, signs, and laboratory test results. TCM group samples were selected based on the criterion that CSF cell count had decreased to below 40/µL, indicating successful treatment. Detailed laboratory test results for patients before and after treatment, along with CM diagnostic findings, are presented in Table 1. Differences in symptoms and laboratory tests before and after treatment are shown in Additional file 4: Table S3. In addition, we recruited 8 patients with non-CNS inflammatory diseases and used their CSF as controls. Due to the extremely low number of cells in their CSF, these 8 patients were randomly divided into two groups. CSF from each group was pooled to form two new samples, designated as non-CNS inflammatory diseases control group (NCID). The UCM, TCM and NCID samples were simultaneously subjected to scRNA-seq and scTCR-seq (Fig. 1A). After filtering and normalization, we performed multistep clustering and constructed cellular maps for a total of 13 samples, encompassing 129,470 cells with an average of 963 genes per cell (Additional file 1: Fig. S1A). Among these cells, 17,794 cells (13.7%) were from the control UCID group, 52,414 cells (40.5%) from the UCM group, and 59,262 cells (45.8%) from the TCM group.

**Table 1:** Demographic data, CSF findings and diagnostic tests results of participants

						UCM					TCM									
No.	Sex	Age	SD	HIV Status	Main complaint	P(mmH_2_O)	WBC(/μL)	Glucose(mmol/L)	Protein(g/L)	G ratio (CSF/BS)	P(mmH_2_O)	WBC(/μL)	Glucose(mmol/L)	Protein(g/L)	G ratio (CSF/BS)	Cul	IIS	CrAg	mNGS	Imaging feature
1	M	65	30	-	Fever	80	225	3.92	0.93	0.54	210	15	3.39	0.27	0.61	+	+	+	C. n	Enhancement of part of the cerebral pia mater
2	F	50	7	-	Headache	120	186	2.95	0.41	0.54	168	39	3.45	0.46	0.57	+	+	+	C. n	Multiple abnormal intracranial enhancements
3	F	40	2	-	Abnormal behavior, unresponsiveness	330	102	0.49	0.9	0.09	175	11	2.8	0.41	0.39	+	+	+	NA	Multiple abnormal intracranial enhancements
4	M	61	14	-	Fever, headache, unresponsiveness	160	110	2.16	2.08	0.16	95	13	2.34	0.79	0.37	-	-	+	C. n	No apparent abnormality
5	M	60	90	-	Recurrent headache	138	111	2.12	2.13	0.29	240	21	3.19	0.44	0.51	+	+	+	NA	Occupying lesion in the right basal ganglia region
6	F	50	7	-	Headache	120	186	2.95	0.41	0.54	170	39	3.39	0.39	0.56	+	+	+	C. n	Multiple abnormal intracranial enhancements

Abbreviation: No., case number; M, male; F, female; SD, symptom duration before admission (days); P, Intracranial pressure; WBC, white blood cell; G, glucose; Cul, culture; IIS, India ink stain; CrAg, cryptococcal antigen; mNGS, metagenomic next-generation sequence; NA, not available; -, negative; +, positive; C.n, *Cryptococcus neoformans*

We identified 5 major cell types: B Cells, Plasma Cells, T and NK Cells, mononuclear phagocytes (MPs), and plasmacytoid dendritic cells (pDCs) (Fig. 1B). This classification was derived from RNA expression profiles of standard marker genes and differentially expressed genes (DEGs) (Fig. 1C). All cells showed high expression levels of housekeeping genes such as *ACTB*, *B2M* and *GAPDH* (Additional file 1: Fig.S1B), confirming the reliability of our data. Using marker gene expression patterns (Additional file 5: Table S4), these major cell types were further subdivided into distinct cell subtypes, including two B cell subtypes, six clusters of CD4^+^ T cells, three subpopulations of CD8^+^ T cells, one subtype of γδ T cells, one proliferating T subtype, one innate lymphocyte subtype (ILC3-RORC), two NK subtypes, four macrophage subtypes, three monocyte subtypes, three dendritic cell subtypes, and three pDC subtypes (Additional file 1: Fig. S1C). Interestingly, the major cell types were conserved across UCM, TCM and NCID samples (Fig. 1D and Additional file 1: Fig. S1D) and among individual patients (Fig. 1E), though their proportions differed. Plasma cells and pDCs exhibited relatively low infiltration levels in all patients. Other immune cell clusters varied between patients, indicating significant heterogeneity in immune cell compositions among CSF samples (Fig. 1F). Comparisons of cell composition between NCID and CM reveal that CSF from CM patients had a significant reduction in MPs, along with marked increase in the populations of B, T and NK cells (Fig. 1F). Additionally, subtype-specific analysis showed a marked increase in the percentage of a specific CD4^+^ T cell subset, terminally differentiated effector memory T cells (CD4^+^ Temra_KLRB1), in CSF of CM patients (Fig. 1G and Additional file 1: Fig.S1E). The proportional distribution of subtypes across each group is shown in Additional file 1: Fig.S2. Based on these observations, we focused our investigation on the role of these altered cell types and their proportions in CM pathogenesis.

### Apoptotic and M2-polarized macrophages in the CSF of CM Patients

**Fig. 2:**
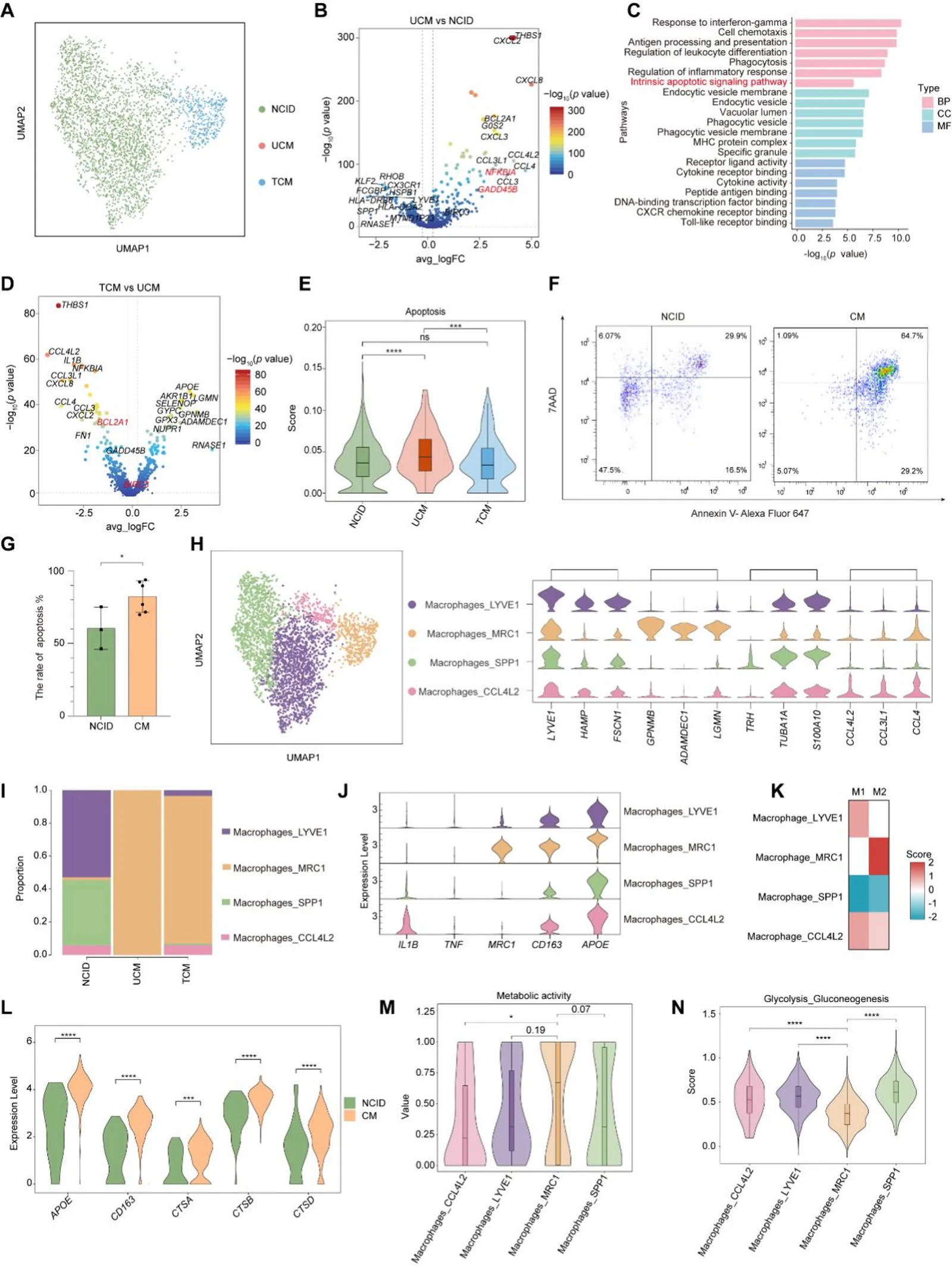
Macrophages in CSF of CM patients exhibit apoptosis and M2 polarization states. **A** Macrophage UMAP plots for different groups. **B** Volcano plot representing differentially expressed genes of macrophages in the UCM and NCID groups. Vertical dotted lines denote cutoffs of 2-fold change, and horizontal dotted lines represent *p* = 0.05 cutoffs. Genes of interest are indicated in red. **C** Significantly enriched Gene Ontology (GO) categories (*p*-value < 0.05) with highly expressed genes of macrophages in UCM group. Results are summarized in three major categories: biological processes (BP, red), cellular components (CC, green), and molecular functions (MF, blue). Horizontal axis indicates value of -log10 (*p*-value), vertical axis indicates most significantly enriched GO terms. Interested GO terms are indicated in red. **D** Volcano plot representing DEGs of macrophages in TCM and UCM groups. **E** Violin/box plots showing apoptotic gene-set scores of macrophages in different groups. ***, *p* < 0.005. *p*-values are determined by the Wilcoxon test. **F**-**G** Validation of macrophage apoptosis in CSF samples from ICM patients and NICD via flow cytometry. F, Representative flow cytometry results for apoptosis. G, Percentages of Annexin V/7-AAD-positive apoptotic cells. Data are presented as the means±SD, *, *p* < 0.05 by unpaired Student’s *t*-test. **H** UMAP plot showing subtypes of macrophages (right) and violin plots showing subset marker expression levels across all discovered clusters of macrophages (left). **I** Stacked bar chart depicting relative proportions of macrophage subtypes in different groups. **J** Violin plots displaying expression levels of selected key macrophage polarization markers across four macrophage clusters. **K** Heatmap showing M1 and M2 scores in four clusters of macrophages. **L** Violin plots illustrating differential expressions of selected macrophage polarization markers in macrophage_MRC1 of NCID and ICM groups. ***, *p* < 0.005. ****, *p* < 0.0001. *p*-values are determined by the Wilcoxon test. **M** Box plot displaying the metabolic strength of different macrophage cell types. *, *p* < 0.05. *p*-values are determined by the Wilcoxon test. **N** Violin plot showing the glycolytic pathway metabolic strength of different macrophages cell types. ****, *p* < 0.0001. *p*-values are determined by the Wilcoxon test.

Macrophages are key components of the innate immune system, playing a crucial role in defending against cryptococcal infection. During the initial cryptococcal infection, alveolar macrophages and dendritic cells form the first line of cellular defense in primary pulmonary cryptococcosis [8]. When macrophages recognize *Cryptococcus* via pattern recognition receptors (PRRs), they phagocytose the fungal pathogen [8]. Subsequently, activated macrophages secrete a variety of cytokines and chemokines to regulate the local immune response against *Cryptococcus* [15, 34]. It is generally agreed that eliminating *Cryptococcus* from the host requires the collaborative action of both innate and adaptive immunity [35]. Given the crucial role of macrophages in the anti-cryptococcal immune response, we focused our investigation on the status of macrophages in CSF of CM patients. The transcriptional landscape of CSF myeloid cells was visualized using UMAP (Fig. 2A). And bootstrap cell proportion differential analyses confirmed a significant reduction in macrophage proportion in UCM group (Additional file 1: Fig. S3A). In contrast, the TCM group showed a significant increase in macrophage levels (Fig. 2A and Additional file 1: Fig.S3A). To explore the mechanisms underlying this difference in macrophage abundance, we conducted differential genes expression (DEGs) analysis between the UCM and NCID groups. Notably, macrophages in the UCM group exhibited upregulated expression of a list of apoptosis-related genes, including *NFKBIA* and *GADD45B* (Fig. 2B) [36, 37]. Furthermore, Gene Ontology (GO) analysis (Fig. 2C) and KEGG analysis (Additional file 1: Fig.S3B) revealed that these DEGs were significantly enriched (*p* < 0.05) in apoptosis pathways, suggesting that *Cryptococcus* may induce programmed cell death in CSF macrophages. Consistently, DEGs analysis between the TCM and UCM groups showed that downregulated expression of apoptosis-related genes in the TCM group, while *BCL2A1* and *BIRC3*, genes that inhibit or regulate apoptosis, were upregulated (Fig. 2D) [38, 39]. Apoptosis gene set scoring analysis further confirmed that the expression of apoptosis-related genes in CSF macrophages of CM patients increased before antifungal treatment and decreased after treatment (Fig. 2E). To further strengthen our observations, we then used flow cytometry to examine macrophage apoptosis in another independent cohort (*n* = 3, NCID; *n* = 6, CM), confirming a higher percentage of apoptotic macrophages cells in the CSF of CM groups (Fig. 2F and G). These data indicate that macrophages in the CSF of CM patients may undergo apoptosis, leading to cell death and a subsequent significant reduction in their numbers.

To investigate the diverse differentiation states and functions of macrophages, we classified them into four distinct subtypes: Macrophages_LYVE1, Macrophages_MRC1, Macrophages_SPP1, and Macrophages_CCL4L2, based on the differential expression of cellular marker genes (Fig. 2H). A notable difference in the proportions of these four macrophage subtypes was observed between the CM and NCID groups (Fig. 2I). After *Cryptococcus* infection, the Macrophages_MRC1 subtype became the dominant macrophage population in the CSF, with other subtypes (Macrophage_SPP1, LYVE1, CCL4L2) showing reduced relative proportions (Fig. 2I). Further analysis of the highly expressed genes in these four macrophage subtypes revealed that Macrophages_MRC1 strongly expressed classical M2 polarization markers (e.g., *MRC1*, *CD163* and *APOE*), while hardly expressing classical M1 polarization markers (e.g., *IL1B* and *TNF*) (Fig. 2J) [40–42]. This is consistent with the elevated M2 scores observed for this subpopulation (Fig. 2K), and the genes used for the M2 score are listed in Additional file 6: Table S5. Moreover, compared with the NCID group, Macrophages_MRC1 in the ICM group highly expressed a range of key molecules critical for M2 polarization, including *APOE*,* CD163*,* CTSA*,* CTSB*, and *CTSD* (Fig. 2L) [40, 41, 43, 44]. These data suggested that *Cryptococcus* may suppress macrophage function, contributing to persist infection.

The metabolic heterogeneity of macrophages significantly influences their function in disease states, such as tumor development or pathogenic infection [45, 46]. To characterize the metabolic profiles of different macrophage subtypes in the CSF of CM patients, we analyzed the expression levels of 50 metabolic pathways (from the KEGG database) across these subtypes. Remarkably, Macrophages_MRC1 showed the highest metabolic activity, with significant enrichment in pathways related to fatty acids, amino acids, nucleotides and lipid metabolism (Fig. 2M and Additional file 1: Fig.S3C). Notably, arginine and proline metabolism were significantly upregulated in Macrophages_MRC1 (Additional file 1: Fig.S3C), potentially due to the high expression of the *SMS* gene encoding a known anti-inflammatory and pro-M2 conversion protein (Additional file 1: Fig.S3D) [47]. These findings indicate that Macrophages_MRC1 are highly metabolically active and M2-polarized, which may relate to their unique functions in CM-infected CSF. In contrast, Macrophages_MRC1 exhibited the lowest glycolytic activity among all subtypes (Fig. 2N). Moreover, glycolysis levels in this subtype were significantly lower in the UCM group and slightly elevated in the TCM group (Additional file 1: Fig.S3E). Since enhanced glycolysis is a key hallmark of M1 macrophages [48], the reduced glycolytic metabolism in Macrophages_MRC1 further supports their M2-polarized identity.

In summary, our findings revealed significant macrophage apoptosis and M2-polarization following infection, supported by metabolic heterogeneity analyses. CSF macrophages have impaired anti-cryptococcal function, hindering pathogen clearance from the brain.

### Increased levels of exhausted CD4^+^ T cells in the CSF of CM Patients

**Fig. 3:**
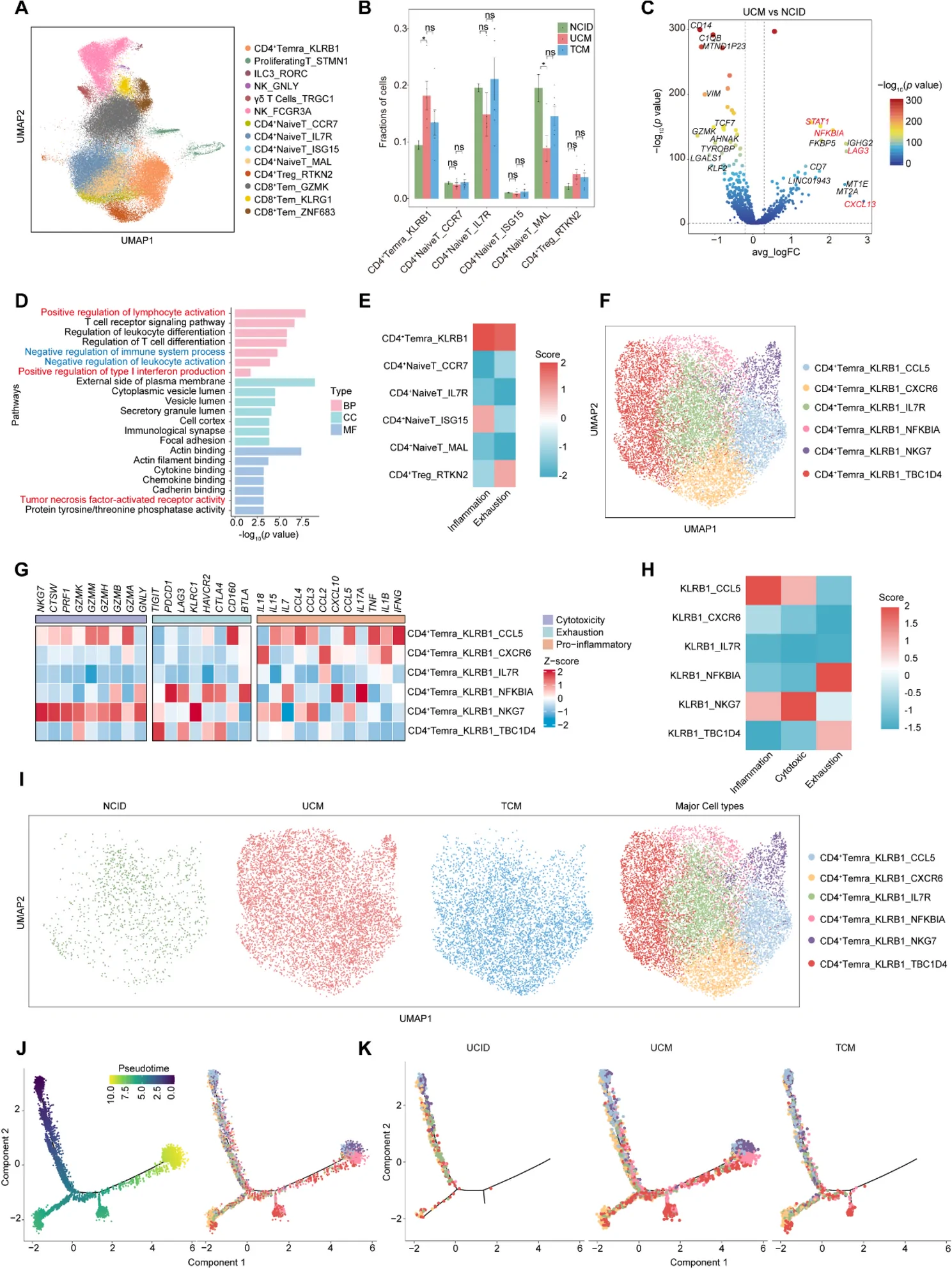
Exhausted CD4^+^ T cells Expand in the CSF of CM Patients. **A** UMAP plot showing the subtype of NK/T cells. **B** Bar graph showing proportion of CD4^+^ subtypes relative to the total T/NK cell population in NCID, UCM, and TCM groups. *, *p* < 0.05. p-values are determined by the Wilcoxon test. **C** Volcano plot representing the DEGs of CD4^+^ Temra_KLRB1 in the UCM and NCID groups. Vertical dotted lines denote cutoffs of 2-fold change, and horizontal dotted lines represent *p* = 0.05 cutoffs. Genes of interest are indicated in red. **D** Significantly enriched GO categories (*p*-value < 0.05) with highly expressed genes of CD4^+^ Temra_KLRB1 cells in the UCM group. Results are summarized in three major categories: biological processes (BP, pink), cellular components (CC, green), and molecular functions (MF, blue). Horizontal axis indicates the value of -log10 (*p*-value), vertical axis indicates the most significantly enriched GO terms. **E** Heatmap showing pro-inflammatory and cell exhaustion gene set expression scores in CD4^+^ cell clusters. **F** UMAP plot showing the subtype of CD4^+^ Temra_KLRB1 cells. **G** Heatmap showing highly expressed genes in the six clusters of CD4^+^ Temra_KLRB1 cells. **H** Heatmap showing the pro-inflammatory, cytotoxicity, and exhaustion gene set expression scores in subtypes of CD4^+^ Temra_KLRB1 cells. **I** CD4^+^ Temra_KLRB1 UMAP plots of different groups. **J** Pseudotime-ordered analysis of CD4^+^ Temra_KLRB1 cells. Cell subtypes are labeled by colors. **K** Pseudotime-ordered CD4^+^ Temra_KLRB1 cells of different groups, including NCID (left), UCM (middle), and TCM (right) groups

Given the significantly higher proportions of NK/T cells in the UCM group compared to the NCID group (Additional file 1: Fig.S1D), and considering the essential role of NK/T cells in clearing *Cryptococcus* infection [13, 49], we subclustered all NK/T cells and identified 14 subsets based on the expression and distribution of canonical NK/T cell markers (Fig. 3A and Additional file 1: Fig.S4A). Analysis of the proportions of these subtypes across the NCID, UCM, and TCM groups revealed a distinct distribution of each cluster among three groups (Additional file 1: Fig.S4B). Notably, the CD4^+^ Temra_KLRB1 cell subset was significantly more abound in the UCM group than that in the NCID group (Fig. 3B), while no such difference was observed for other NK/T subtypes (Additional file 1: Fig.S4C). To explore the characteristics of CD4^+^ Temra_KLRB1 cells, we compared their expression profiles between the UCM and NCID groups. Interestingly, CD4^+^ Temra_KLRB1 cells in the UCM group showed a unique gene expression pattern, with upregulation of both pro-inflammatory genes (e.g., the Th1 master transcription factor *STAT1* and the chemokine *CXCL13*) and anti-inflammation/exhaustion-related genes (e.g., *NFKBIA* and *LAG3*) (Fig. 3C) [50–53]. GO enrichment analysis indicated that CD4^+^ Temra_KLRB1 cells in the UCM group were associated with both positively and negatively regulated inflammatory pathways (Fig. 3D). This was consistent with the elevated expression scores of pro-inflammatory and cell exhaustion gene sets in this subpopulation (Fig. 3E), suggesting that these cells are in a state of both activation and exhaustion.

The contradictory properties of CD4^+^ Temra_KLRB1 raise the intriguing possibility of further subdivision into more refined subtypes. By adjusting the resolution parameter in the Seurat R package, we identified six transcriptionally distinct subtypes, which are visualized as separate clusters in the UMAP projection (Fig. 3F). These subtypes were annotated as CD4^+^ Temra_KLRB1_CCL5, CD4^+^ Temra_KLRB1_CXCR6, CD4^+^ Temra_KLRB1_IL7R, CD4^+^ Temra_KLRB1_ NFKBIA, CD4^+^ Temra_KLRB1_NKG7, and CD4^+^ Temra_KLRB1_TBC1D4 based on their signature genes (Additional file 1: Fig.S4D). To explore the functional characteristics of each subtype, we conducted transcriptional profiling analysis (Fig. 3G). CD4^+^ Temra_KLRB1_CCL5 predominantly expressed pro-inflammatory genes, including *IFN-γ* and various chemokine ligands. In contrast, CD4^+^ Temra_KLRB1_NFKBIA and CD4^+^ Temra_KLRB1_TBC1D4 showed a tendency towards inhibiting inflammation and exhaustion, as evidenced by the prominent expression of genes such as *LAG3* and *CTLA4* [53, 54]. Interestingly, CD4^+^ Temra_KLRB1_CXCR6 and CD4^+^ Temra_KLRB1_IL7R appeared to be relatively quiescent subtypes, with fewer highly expressed genes compared to the others. Notably, CD4^+^ Temra_KLRB1_NKG7 was identified as a subset of cytotoxic T cells, characterized by high expression of granzyme-encoding genes like *GZMB* and *GZMK* (Fig. 3G). Consistently, the scoring of inflammation, cytotoxicity and exhaustion gene sets for each CD4^+^ T cell type further supported the findings from the transcriptomic analysis (Fig. 3H). CD4^+^ Temra_KLRB1_CCL5 showed higher inflammation scores than the other subsets, while CD4^+^ Temra_KLRB1_NKG7 exhibited the highest cytotoxic state. Meanwhile, CD4^+^ Temra_KLRB1_NFKBIA and CD4^+^ Temra_KLRB1_TBC1D4 had higher exhaustion scores than the other subsets (Fig. 3H), indicating that these cell subtypes may be in a highly exhausted state with functional impairment. More importantly, distribution of different CD4^+^ Temra_KLRB1 subsets across groups (Fig. 3I and Additional file 1: Fig.S4E) identified that the majority of CD4^+^ Temra_KLRB1_NFKBIA and a significant proportion of CD4^+^ Temra_KLRB1_TBC1D4, subtypes characterized by T cell exhaustion, were predominantly found in the UCM group (combined with Fig. 3F), highlighting that these cell subsets may play a role in the protracted and recalcitrant nature of CM disease.

To decipher the intricate dynamic immune states and cellular transitions of CD4^+^ Temra_KLRB1 cells in the CSF of CM patients, we used the monocle2 R package to construct a pseudo-timeline for individual cells. This analysis revealed that the CD4^+^ Temra_KLRB1_CCL5 predominantly occupied the starting point of the trajectory, while CD4^+^ Temra_KLRB1_NFKBIA and CD4^+^ Temra_KLRB1_NKG7 were mainly located at the terminal state (Fig. 3J). Specifically, the transition of CD4^+^ Temra_KLRB1 cells was found to initiate with a proinflammation state (characterized by CD4^+^ Temra_KLRB1_CCL5 cells) and ultimately reach an exhausted state (characterized by CD4^+^ Temra_KLRB1_NFKBIA and CD4^+^ Temra_KLRB1_NKG7 cells) (Additional file 1: Fig.S4F). To further clarify the transition states of CD4^+^ Temra_KLRB1 cells in different patient groups, we analyzed their trajectories in the NCID, UCM and TCM groups separately. Notably, cells in the NCID and TCM groups were predominantly at the initial stage of the trajectory path, whereas a large number of exhausted cells at the terminal states were observed in the UCM group (Fig. 3K and Additional file 1: Fig.S4G). This collectively depicts the shift from pro-inflammatory to exhausted cells following cryptococcal infection of the CNS.

Taken together, our results suggest that CD4^+^ T cell subsets in CM patients exhibit strong exhaustion and suppressed inflammation.

### Clonal expansion of T cells and preferential V(D)J gene usage in CM patients

**Fig. 4:**
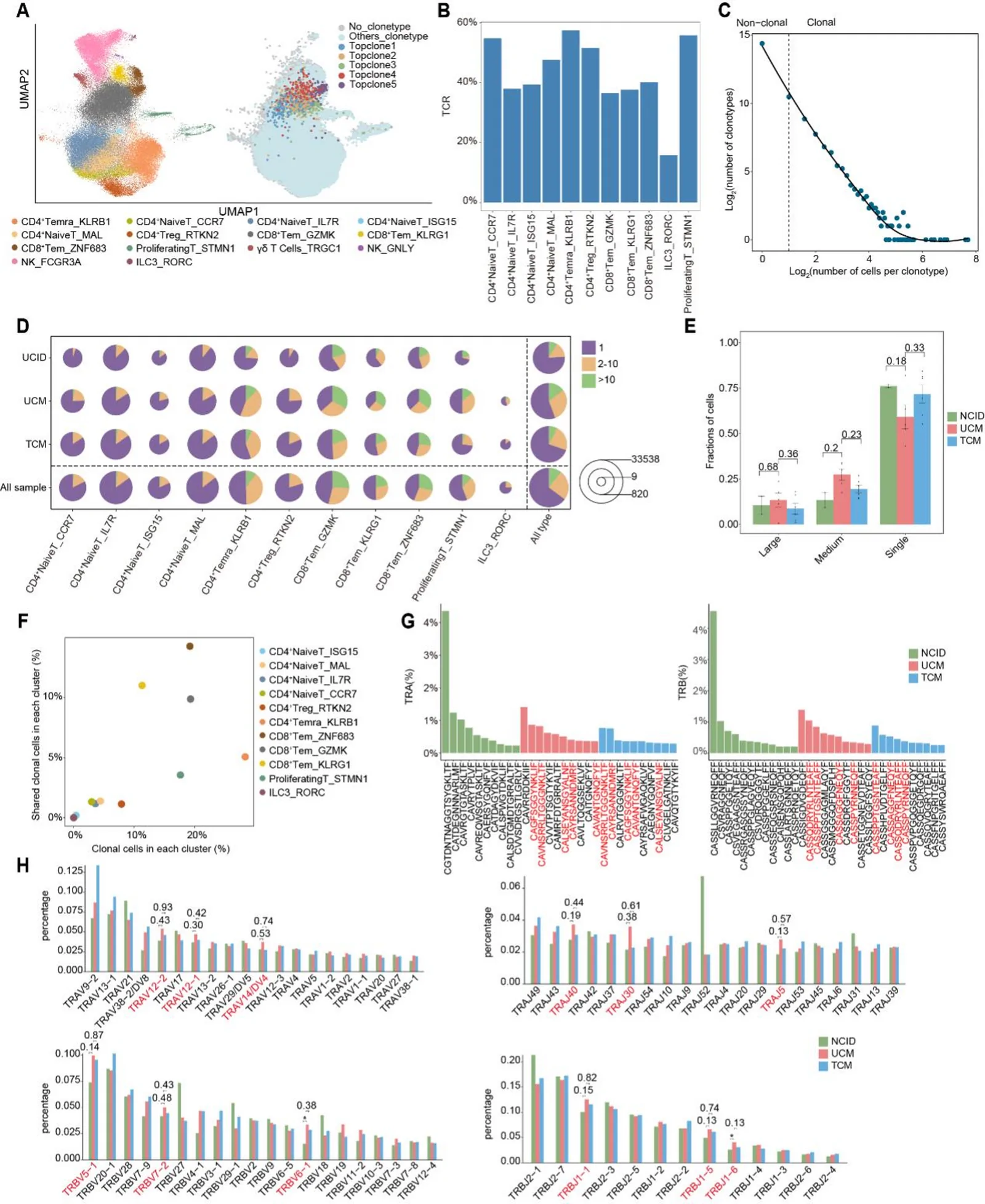
Clonal expansion of T cells and preferred usage in V(D)J genes in CM patients. **A** UMAP of T cells derived from CSFs. Clusters are denoted by colors labeled with inferred cell types (left) and TCR detection (right). **B** Bar plots showing percentage of TCRs detected in each T cell subtype. **C** Correlation between the number of T cell clones and the number of cells of each clonal type, with the dashed line separating the non-clonal cells from the clonal cells. Solid line shows negative correlation between the two axes via LOESS fitting. **D** Clonal statuses of different T cell subtypes and percentages of different levels of clonal T cells across three groups. Sizes of the circles represent total number of clonotypes. **E** Comparison of clonal statuses of T cells in three groups. Criteria for clone status grouping: Single for 1 clone; Medium for 2-10; Large for >10. *p*-values are determined by the Wilcoxon test. **F** Comparison between fractions of clonal cells in each subtype (x axis) and percentages of cells with TCRs shared across clusters (y axis). **G** Top ten CDR3 usages across three groups. Shared CDR3 sequences are indicated in red. **H** Usage of some TRAV (top left), TRAJ (bottom left), TRBV (top right), and TRBJ (bottom right) genes across three groups. *, *p* < 0.05. *p*-values are determined by the unpaired two-tailed Student’s *t*-test

T cell clonal expansion and V(D)J recombination play fundamental roles in adaptive immunity, particularly in response to infection [55]. Thus, we reconstructed T cell receptor (TCR) sequences from TCR sequencing data (Additional file 7: Table S6) to explore the clonal relationship among individual T cells and the usage of V(D)J genes across the three groups. Firstly, approximately 40%-60% of cells in all subsets were matched with TCR information, except for the NK subset and ILC3-RORC (Fig. 4A, B). Compared to the NCID group, clonal expansion was evident in CM patients (Fig. 4C-E). Moreover, the extent of clonal expansion in the UCM group was greater than that in the TCM group (Fig. 4D, E), indicating that T cell clonal expansion occurs after cryptococcal infection, while the proportion of clonally expanded T cells decreased after treatment. This is consistent with the improved clinical manifestations of the patients (Table 1). We also observed varying degrees of clonal expansion among T cell subsets (Fig. 4D). Notably, effector T cell subsets, including CD4^+^ effector-KLRB1, CD8^+^ effector-GZMK, CD8^+^ effector-KLRG1, CD8^+^ effector-ZNF683 and proliferating-STMN1, exhibited high proportions of clonal cells (Fig. 4A and D and Additional file 1: Fig.S5A) and high proportions of inter-cluster clonal cells (Fig. 4F), suggesting dynamic state transitions in effector T cells.

Next, we compared V(D)J gene usage across the three groups to characterize the dynamics and gene preference of TCRs in CM and NCID (Fig.G-H and Additional file 1: Fig. S5B). Among the three groups, the top 10 complementarity determining region 3 (CDR3) sequences differed, but the UCM group and TCM groups shared some CDR3 sequences due to paired patient samples (Fig. 4G). Moreover, the usage percentage of the top 10 CDR3 sequences in the NICD group was lower and more balanced compared to the UCM and TCM groups (Fig. 4G). Notably, we identified distinct V(D)J gene usage with increased diversity in CM patients, observed across multiple T-cell subsets for both TRA and TRB genes (Fig. 4H, Additional file 1: Fig.S5B and Additional file 1: Fig.S6). We also found that the UCM group had a higher representation of TRAV12-2, TRAV12-1, TRAV14-DV4, TRAJ40, TRAJ30 and TRAJ5 compared to the TCM and NCID groups, while the preferred TRBV gene in the UCM group were TRBV5-1, TRBV7-2, TRBV6-1, TRBJ1-1, and TRBJ1-5, TRBJ1-6 (Fig. 4H). This selective V(D)J gene usage suggests that different immunodominant epitopes may drive the molecular composition of T cell responses and could be associated with *Cryptococcus*-specific infection.

### A constrained role of B cells in the CSF of CM patients

**Fig. 5:**
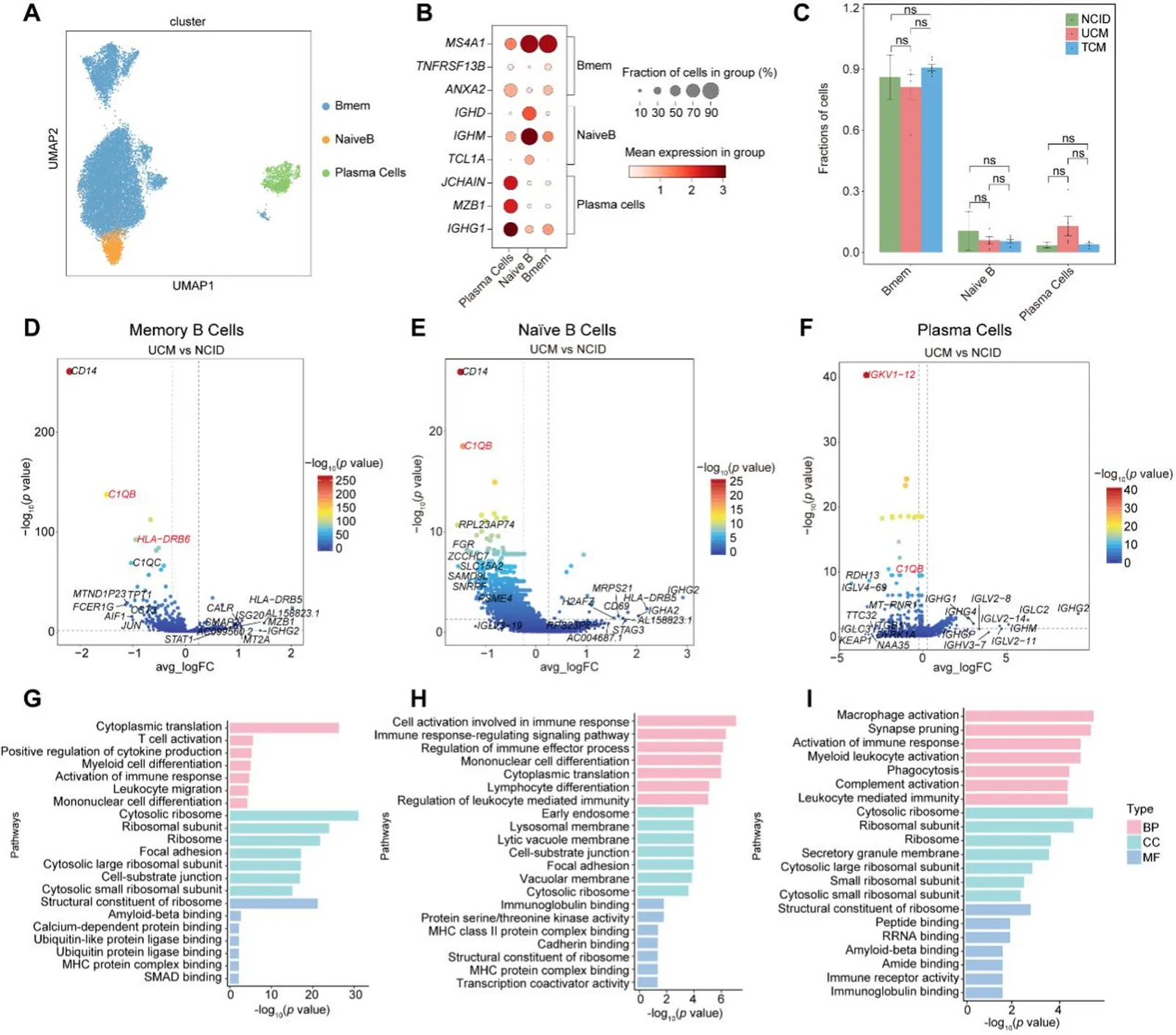
B cells exhibit a constrained role in the CSF of CM patients. **A** UMAP plot showing the subtype of B cells. **B** Bubble plot showing marker genes for each cell cluster. Color intensity denotes average expressions. Dot sizes indicate the percentages of cells expressing the gene. **C** Bar graph showing the proportion of B subtypes relative to the total B cell population in NCID, UCM, and TCM groups. p-values are determined by the Wilcoxon test. **D**-**F** Volcano plot representing the DEGs of memory B cells (**D**), naïve B cells (**E**), and plasma cells (**F**) in UCM and NCID groups. Vertical dotted lines denote cutoffs of 2-fold change, and horizontal dotted lines represent *p* = 0.05 cutoffs. Genes of interest are indicated in red. (**G**-**I**) Significantly enriched GO categories (*p*-value < 0.05) with downregulated DEGs of memory B cells (**G**), naïve B cells (**H**), and plasma cells (**I**) between UCM and NCID groups. Results are summarized in three major categories: biological processes (BP, red), cellular components (CC, green), and molecular functions (MF, blue). Horizontal axis indicates value of -log10 (*p* value), and vertical axis indicates the most significantly enriched GO terms.

We then clustered B cells and identified three distinct subtypes: memory B cells, naïve B cells, and plasma cells (Fig. 5A). These subtypes each have unique marker gene expression profiles (Fig. 5B). Analysis of the proportions of these subtypes across the NCID, UCM and TCM groups revealed no significant differences in their distribution (Fig. 5C). To explore functional alterations in these B cell subtypes, we performed DEGs analysis between groups. Interestingly, regardless of the specific subtype or treatment stage, the number of upregulated genes was relatively small compared to the NCID group, while a large number of genes were downregulated (Fig. 5D-F). For example, we observed a marked downregulation of *C1QB* in both B cells and plasma cells after CM infection. As a key mediator of the classical complement cascade, *C1QB* initiates C3 cleavage to generate C3d, which binds CR2 to enhance B cell receptor (BCR) signaling. Reduced *C1QB* expression likely impairs B cell activation and function [56, 57], and may also disrupt complement-dependent pathogen opsonization and reduce microbial clearance [58]. In memory B cells (Bmem), transcripts encoding *HLA-DRB1* and other MHC class II molecules were significantly suppressed. Given the critical role of MHC II in antigen presentation and CD4⁺ T cell priming, decreased *HLA-DRB1* expression (Additional file 1: Fig.S7A) may hinder adaptive immune engagement and promote an immunosuppressive environment. Concurrently, plasma cells showed significant downregulation of *IGKV1-12*, implying defects in κ light-chain synthesis that could directly impair antibody assembly and secretion, weakening the humoral response against CM [59]. Notably, compared with the UCM group, *C1QB* expression was elevated in TCM group, suggesting the recovery of B cell-mediated immune function beneficial for pathogen clearance (Additional file 1: Fig.S7B-C). Enrichment analysis of downregulated genes in the UCM group compared to the UCID group showed that all three subtypes had downregulation of immune cell activation and pro–inflammatory pathways, including T cell activation, immune response–regulating signaling pathways. Notably, the downregulated gene set in plasma cells was enriched for complement activation pathways, consistent with the observed dysregulation of the classical complement cascade (Fig. 5G-I). These findings suggest that the ability of the humoral immune response to neutralize antigens may be compromised after cryptococcal infection.

### The CCL3-CCR5-dependent interplay between activated Macrophages_MRC1 and CD4^+^ Temra_KLRB1 cells in CSF of CM patients

**Fig. 6:**
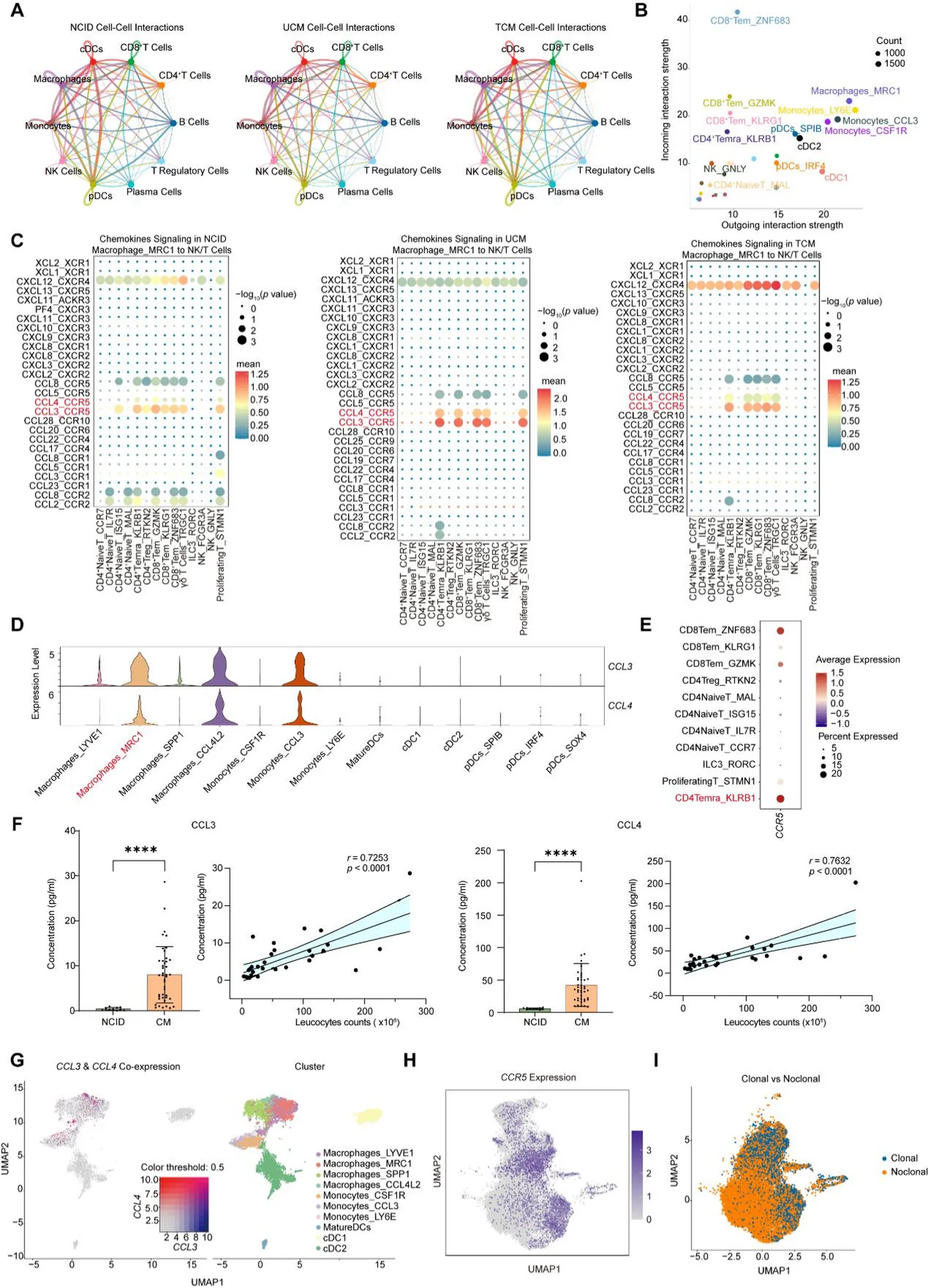
Activated Macrophages_MRC1 cells communicate with CD4^+^ Temra_KLRB1 cells via CCL3-CCR5 in CM CSF. **A** Network plots showing CSF immune signaling pathways in NCID, UCM, and TCM groups, where selected subtypes (e.g., CD4^+^ T cells) are merged. **B** Scatter plot showing intensities of cellular communication. Horizontal axis denotes outgoing interactions, and the vertical axis denotes incoming interactions. **C** Dot plot indicating signaling molecules between ligand/receptor-expressing cells among different groups. **D** Violin plots indicating cell types that express CCL3 and CCL4 in the CSF. **E** Bubble plot showing expressions of CCR5 for each cell cluster. Color intensity denotes average expression. Dot sizes denote percentages of cells expressing the genes. **F** Validation of the concentration of CCL3 (left) and CCL4 (right) in the NCID and ICM groups using Luminex liquid suspension chips. ****, *p* < 0.0001. p-values are determined by the Mann-Whitney *U* test. Correlation between CSF leucocyte counts (x10^6^/L) and CCL3/4 levels in ICM, respectively. **G**-**H** UMAPs showing expression of CCL3/4 by myeloid cells and their subtypes (**G**) and CCR5 by T cells (**H**). **I** Distribution of clonal and nonclonal CSF T cells.

Intercellular communication is key for immune cell activation, chemotaxis, and regulatory functions [60]. Using CellChat (version 0.0.2), a tool that leverages a database of ligand-receptor interactions to analyze intercellular communication networks from single-cell RNA sequencing data [33], we explored communication between cell clusters in CSF samples, focusing on their unique patterns and ligand-receptor signals. Across the three groups, the overall intercellular interaction patterns were quite similar (Fig. 6A). Our analysis identified 89 signaling pathways, including MHC-I, MHC-II and GALECTIN pathways (Additional file 1: Fig.S8A). A scatter plot of cell communication strength (Fig. 6B) shows that each cell type varies in its strength as a receptor or ligand-expressing cell. Notably, CD4^+^ Temra_KLRB1 cells and CD8^+^ T cells have the strongest communication activity as receptor cells. Certain mononuclear phagocytes, including Macrophages_MRC1, Monocytes_LY6E, and Monocytes_CCL3, exhibit the strongest signals as ligand-expressing cells.

To identify disease-relevant signaling pathways, we compared signaling pathways across the NCID, UCM and TCM groups. A key finding was that the interaction between C-C Motif Chemokine Ligand 3 (CCL3/4) and C-C Motif Chemokine Receptor 5 (CCR5), occurring between Macrophages_MRC1 and CD4^+^ Temra_KLRB1 cells, was significantly upregulated in the UCM group compared to the NCID group. Importantly, this interaction was substantially reduced after treatment (in the TCM group), with levels nearing those in control samples (Fig. 6C), suggesting it may play a role in disease-associated cell communication. CCR5 is a surface chemotactic receptor for inflammatory CC-chemokines like CCL3, CCL4 and RANTES, and it regulates T cell migration to the infection site [61]. We analyzed the expression of CCL3/4 and CCR5 across CSF cell types and the results showed that CCL3 and CCL4 were expressed by Macrophages_MRC1, Macrophages_CCL4L2, and Monocytes_CCL3 (Fig. 6D and G), while CCR5 was expressed by CD4^+^ T_KLRB1, CD8^+^ T_ZNF683, and CD8^+^ T-GZMK (Fig. 6E). Luminex assays showed higher levels of CCL3 and CCL4 in the CSF of CM patients compared to NCID (Fig. 6F). Notably, CCL3 and CCL4 levels strongly positively correlated with CSF leukocyte counts (*r* = 0.7253, *p* < 0.0001; *r* = 0.7632, *p* < 0.0001) (Fig. 6F) and negatively correlated with CSF/blood glucose ratio (*r* = -0.7060, *p* < 0.0001; *r* = -0.6959, *p* < 0.0001) (Additional file 1: Fig. S8B). These correlations suggest they could serve as indicators of intracranial infection burden and biomarkers of neuroinflammatory progression. Additionally, TCR sequencing results revealed an association between CCR5 expression and clonal T cells (Fig. 6H and I). Taken together, these findings indicate that myeloid cells communicate with T cells via the CCL3/CCL4-CCR5 in the CSF of CM patients.

## Discussion

CM is a major public health challenge, with high morbidity and mortality, especially in resource-limited areas [62, 63]. *Cryptococci* are ubiquitous encapsulated yeasts and several species are classified as “critical threat” on the World Health Organization (WHO) list of fungal priority pathogens [64]. This designation highlights the urgent need for more research into their pathophysiology and the development of new anti-fungal therapies. Notably, the *Cryptococcus neoformans* and *Cryptococcus gattii* species complexes are the primary causes of human diseases, with *C. neoformans* being the leading etiologic agent of CM in immunocompromised individuals, most prominently in HIV-positive patients [8]. Despite the availability of antifungal therapies, clinical outcomes remain poor, attributable to diagnostic delays, limited access to treatment, and complications such as elevated intracranial pressure and immune reconstitution inflammatory syndrome (IRIS). Early diagnosis, timely antifungal intervention, and meticulous management of intracranial pressure are critical for improving survival. Given its substantial morbidity and mortality, CM represents a pressing global public health concern, underscoring the need for improved diagnostic tools, optimized therapeutic strategies, and robust preventive measures. Here, we used the unbiased scRNA-seq approach to understand the unique human immune compartment and identify mechanism locally driving CM disease progression in patients with non-HIV CM.

Our datasets allow detailed analysis of immune responses in CM patients. A key finding is the significant reduction in mononuclear phagocytes (MPs), including macrophages and dendritic cells, in the CSF of CM patients compared to NCID controls. This decrease is mainly due to increased apoptosis in the UCM group, as shown by upregulated apoptosis-related genes (e.g., *NFKBIA* and *GADD45B*) in macrophages [36, 37]. This suggests that *Cryptococcus* invasion of the CNS triggered programmed cell death in these cells. Remaining macrophages in CM patients have a distinct M2-polarized phenotype, with high expression of *MRC1* and *CD163*, and a metabolic profile (reduced glycolysis and enhanced arginine metabolism) typical of anti-inflammatory M2 macrophage state [41]. Notably, Macrophages_MRC1 exhibited a characteristic of perivascular macrophages (PVMs)—a specialized subset of border-associated macrophages (BAMs)—defined by high expression of *MRC1*, *CD163*, and *FOLR2*, coupled with an absence of microglial (*TMEM119*, *P2RY12*) and monocyte-derived markers (*CD14*, *FCGR3A*) [65]. Given their role as frontline sentinels at the blood-brain interface, the pronounced apoptosis and M2 polarization of these PVMs in UCM patients suggest that *Cryptococcus neoformans* specifically incapacitates this vascular defense barrier, thereby compromising CNS immune surveillance and facilitating fungal persistence. Another important finding is the altered T cell composition and functions in the CSF of CM patients. We identified a specific CD4^+^ T cell subtype (CD4^+^ Temra_KLRB1), which is more abundant in the UCM group than in NCID controls. These cells have a dual transcriptional profile, expressing both pro-inflammatory and exhaustion-related genes. Further subdivision revealed subtypes, some pro-inflammatory while others exhausted (with upregulated inhibitory receptors like *LAG3*) [52, 53]. Pseudotime analysis supports a dynamic transition, where pro-inflammatory CD4^+^ T cells differentiate into exhausted subsets. Additionally, cytotoxic CD4^+^ T cells (high expression of *NKG7*, *GZMB*, and *GZMK*) emerged, possibly an adaptive attempt to fight the fungus via direct cytotoxicity. In contrast, B cells play a limited role in the humoral immune response in CM patients. Across naïve, memory, or plasma B cell subtypes, genes related to complement activation, immune cell activation and pro-inflammatory pathways, are significantly downregulated in the UCM group compared to NCID controls. This suggests that the ability of B cells to mount an effective antibody response against *Cryptococcus* is compromised in the context of CM. This aligns with murine studies showing B cells do not protect against *C. neoformans* [66], and our work adds that B cells enter a functionally suppressed state post-infection and restoring their function could be a new therapeutic strategy.

Importantly, CD4^+^ Temra_KLRB1 cells and Macrophages_MRC1 cells show increased CCL3/CCL4-CCR5 signaling in the UCM group, which decreased after antifungal treatment. CCR5, a well-studied chemokine receptor, regulates T-lymphocyte migration to infection sites by binding CCL3/4 [67]. Luminex assays confirmed high CCL3/4 levels in the CSF of UCM group, with correlations to CSF leukocyte counts (indicating infection burden) and CSF/blood glucose ratio (indicating neuroinflammation). CCR5 is also associated with clonally expanded T cells, suggesting it regulates antigen-specific T cell homing to CSF via myeloid cell-derived CCL3/4. Notably, CCR5 is an HIV-1 co-receptor, and HIV-positive patients have high CM rates (19–68%) [68, 69], making CCR5 a potential target for CM and HIV treatment.

This study has limitations. First, obtaining sufficient immune cells proved highly challenging, as the CSF of NCID patients is characterized by an acellular profile, containing rare lymphocytes and monocytes. Second, the patient sample size is relatively small, which may limit the generalizability of our findings. Furthermore, we acknowledge that our analysis captures patients at an advanced stage of established infection; thus, conclusions regarding the early initiation of pathogenesis must be drawn cautiously, as the exact temporal progression of immune dysregulation cannot be fully resolved in human studies. Third, this study relies exclusively on human data, and our conclusions were primarily derived from bioinformatic analyses that depend on the underlying assumptions of the algorithms used. Additionally, there is a lack of functional evidence to validate key observations. Ideally, future studies involving longitudinal samples from a larger cohort of CM patients, coupled with in vivo animal experiments, would help clarify the causal relationships between the immune characteristics of distinct cell types and the pathological mechanisms driving the disease.

## Conclusions

In this study, our comprehensive single-cell transcriptomic analysis of CSF from CM patients, combined with an exploration of the complex immune responses to *Cryptococcus* infection, has revealed multifaceted dysregulation of the immune landscape within the CNS. Specifically, impaired macrophage function, the dynamic shift of T cells from a pro-inflammatory to an exhausted state, and compromised B cell activation collectively contribute to the protracted and recalcitrant nature of CM. Furthermore, the identified CCL3/CCL4-CCR5 signaling axis represents a promising therapeutic target for future interventions aimed at restoring the host’s anti-cryptococcal immune response.

## Supplementary Information

Below is the link to the electronic supplementary material.

Supplementary Material 1: Additional file 1: Fig. S1 to S8

Supplementary Material 2: Additional file 2: Table S1

Supplementary Material 3: Additional file 3: Table S2

Supplementary Material 4: Additional file 4: Table S3

Supplementary Material 5: Additional file 5: Table S4

Supplementary Material 6: Additional file 6: Table S5

Supplementary Material 7: Additional file 7: Table S6

## Data Availability

The authors declare that the data supporting the findings of this study are available within the article and its Supplementary Information files. The ScRNA-seq and TCR-seq data have been deposited in the NCBI Sequence Read Archive under accession number GSE302409. Other relevant data supporting the findings of this study are available within this article and its associated Supplementary material.

## Abbreviations

CM: Cryptococcal meningitis
UCM: untreated cryptococcal meningitis
TCM: treated cryptococcal meningitis
ICM: integrated cryptococcal meningitis
NCID: non-CNS inflammatory diseases
CNS: central nervous system
scRNA-seq: single-cell RNA sequencing
scTCR-seq: single-cell T cell receptor sequencing
CrAg: Cryptococcal Antigen
CCR5: C-C motif chemokine receptor 5
CCL3: C-C motif chemokine ligand 3
CCL4: C-C motif chemokine ligand 4
CXCL13: C-X-C motif chemokine ligand 13
