# Supplementary material for: Single-cell immune landscape of cerebrospinal fluid in non-HIV cryptococcal meningitis
Source: BMC Med. 2026 Aug 10;24:627. doi: 10.1186/s12916-026-05142-x (PMC13746546; doi:10.1186/s12916-026-05142-x)
Supplement: Supplementary file 1 [file 12916_2026_5142_MOESM1_ESM.docx]

**Supple****mental** **Figure and Figure legends**

**Fig.S1.** **scRNA-seq revealed the immune landscape of CM CSF.**

(A) Histogram indicating the total number of mapped clean reads (left panel) and the number of detected genes (right panel). (B) Violin plot showing the expression levels of 8 known housekeeping genes, in all cells. (C) UMAP plot of subtype of cell clusters. (D) Bar graph showing the proportion of each cell type in NCID group, UCM group, and TCM group. *, *p* < 0.05. **, *p* < 0.01 *p* values were determined by Wilcoxon test.

(E) Stacked bar chart depicting relative proportions of all subtypes in different groups.

**Fig.S2. Proportional distribution of cell subtypes across each group.**

(A-D) Bar graph displaying the fraction of cells for distinct immune subtypes within the MPs (A), T cell/NK cell (B), pDC (C), and B cell (D) lineages, stratified by diagnosis (NCID, UCM, TCM). Data represent the percentage of total cells within each sample. *, *p* < 0.05, *p* values were determined by Wilcoxon test.

**Fig.S3.** **Macrophages in the CSF of CM patients exhibited apoptosis and M2 polarization states.**

(A) Bar graph showing the proportion of macrophages in NCID group, UCM group, and TCM group. ***, *p* < 0.005. *p* values were determined by Wilcoxon test. (B) KEGG enrichment analysis of highly expressed genes of macrophages in UCM group. KEGG gene sets were used to perform the pathway enrichment analysis (Methods). Only selected pathways are shown. (C) Bubble plot showing the metabolic activity analysis of different macrophage subtypes. The circle size and color darkness both represent the scaled metabolic score. (D) Violin plots displaying the expression levels of *SMS* across the four macrophage clusters. (E) Bubble plot showing the metabolic activity analysis of different macrophage subtypes among different groups. The circle size and color darkness both represent the scaled metabolic score.

**Fig.S4.** **Exhausted CD4^+^ T cells Are Increased in the CSF of CM Patients**

(A) Violin plots showing subset marker expression levels across all discovered clusters of NK/T cells. (B) Stacked bar chart depicting the relative proportions of NK/T cell types in different groups. (C) Bar graph showing the proportion of NK/CD8^+^ T cell types in NCID group, UCM group, and TCM group. *, *p* < 0.05. *p* values were determined by Wilcoxon test. (D) Violin plots showing subset marker expression levels across all discovered clusters of CD4^+^ Temra_KLRB1 cells. (E) Stacked bar chart depicting the relative proportions of CD4^+^ Temra_KLRB1 cell types in different groups. (F) The pseudotime plot showing the dynamics of pro-inflammation (left panel), cytotoxicity (middle panel) and exhausted signals (right panel) in CD4^+^ Temra_KLRB1 cells. (G) Pseudotime-ordered analysis of CD4^+^ Temra_KLRB1 cells. Different groups are labeled by colors.

 **Fig.S5.** **Clonal expansion of T cells and preferred usage in V(D)J genes in CM patients**.

(A) The distribution of the clone state of T cells in each group. (B) TRA/B rearrangement differences across three groups. The colors indicate the usage percentage of specific TRAV/J and TRBV/D/J genes.

**Fig.S6. TRA/B rearrangement differences across three groups.** The colors indicate the usage percentage of specific V-J gene pairs.

**Fig.S7. Differential genes between groups in B cells.**

(A-C) The volcano plot represents the DEGs of memory B cells (A), naïve B cells (B), and plasma cells (C) between the UCM and the TCM group. Vertical dotted lines denote the cutoff of 2-fold change, and the horizontal dotted line represent the *p* = 0.05 cutoff. (D) Violin plots displaying the expression levels of *HLA-DRB1* between the NCID and UCM group. ****, *p* < 0.0001. *p* value was determined by Wilcoxon test.

**Fig.S8. Intercellular communication and CCL3/4 correlation with CSF/blood glucose ratio.**

(A) Heatmap delineating signaling pathways exhibiting the highest contribution to outgoing or incoming signaling among cell subtype populations. (B) Correlation analysis between the CSF/blood glucose ratio and CCL3/4 levels in CM.
