# Supplementary material for: Single-cell immune landscape of cerebrospinal fluid in non-HIV cryptococcal meningitis
Source: BMC Med. 2026 Aug 10;24:627. doi: 10.1186/s12916-026-05142-x (PMC13746546; doi:10.1186/s12916-026-05142-x)
Supplement: Supplementary file 2 [file 12916_2026_5142_MOESM2_ESM.docx]

**Table S1** Demographic data, CSF findings, diagnostic tests results, and final diagnosis of participants of flow cytometry.

| No. | Sex | Age | HIV Status | SD | Main complaint | P (mmH_2_O) | WBC  (/μL) | Protein  (mg/L) | Glucose (mmol/L) | CSF/blood glucose ratio | IIS | Cul | CrAg | mNGS | Final diagnosis |
| --- | --- | --- | --- | --- | --- | --- | --- | --- | --- | --- | --- | --- | --- | --- | --- |
| 1 | F | 70 | - | 30 | Memory impairment | 110 | 15 | 2500 | 0.06 | NA | - | + | + | NA | CM |
| 2 | F | 71 | - | 117 | Recurrent headache with vomiting | 160 | 45 | 740 | 3.06 | 42.68 | - | - | + | NA | CM |
| 3 | F | 70 | - | 30 | Memory impairment | 145 | 23 | 670 | 0.67 | 10.68 | - | + | + | NA | CM |
| 4 | M | 72 | - | 40 | Recurrent headache | 100 | 49 | 3570 | 1.22 | NA | + | - | + | NA | CM |
| 5 | M | 41 | - | 68 | Headache | 120 | 37 | 1520 | 0.61 | 8.24 | + | - | + | NA | CM |
| 6 | F | 71 | - | 117 | Recurrent headache with vomiting | 120 | 49 | 660 | 3.86 | 40.76 | - | - | + | NA | CM |

Abbreviation: No., case number; M, male; F, female; HIV, Human Immunodeficiency Virus; SD, symptom duration before admission (days); P, Intracranial pressure; WBC, white blood cell; IIS, India ink stain; Cul, culture; CrAg, cryptococcal antigen; mNGS, metagenomic next-generation sequence; NA, not available; -, negative; +, positive; CM, cryptococcal meningitis.
