# Supplementary material for: Single-cell immune landscape of cerebrospinal fluid in non-HIV cryptococcal meningitis
Source: BMC Med. 2026 Aug 10;24:627. doi: 10.1186/s12916-026-05142-x (PMC13746546; doi:10.1186/s12916-026-05142-x)
Supplement: Supplementary file 3 [file 12916_2026_5142_MOESM3_ESM.docx]

**Table S2** Demographic data, CSF findings, diagnostic tests results, and final diagnosis of participants for Luminex cytokine analysis.

| No. | Sex | Age | HIV Status | SD | Main complaint | P (mmH_2_O) | WBC  (/μL) | Protein  (mg/L) | Glucose (mmol/L) | CSF/blood glucose ratio | IIS | Cul | CrAg | mNGS | Final diagnosis |
| --- | --- | --- | --- | --- | --- | --- | --- | --- | --- | --- | --- | --- | --- | --- | --- |
| 1 | M | 66 | - | 10 | Fever, headache | 180 | 140 | 1000 | 1.37 | 22.46 | + | + | - | *C.n* | CM |
| 2 | M | 71 | - | 10 | Headache | >300 | 153 | 760 | 1.11 | 15.59 | + | + | - | *C.n* | CM |
| 3 | M | 47 | - | 14 | Recurrent fever with headache | 270 | 91 | 1180 | 1.11 | 21.06 | + | + | - | - | CM |
| 4 | F | 36 | - | 7 | Recurrent headache | 320 | 110 | 2980 | 1.57 | 31.46 | + | + | + | *C.n* | CM |
| 5 | F | 67 | - | 10 | Fever with headache | 70 | 364 | 1740 | 2.06 | 30.03 | + | - | - | *C.n* | CM |
| 6 | M | 62 | - | 60 | Headache, unconsciousness | 120 | 26 | 540 | 3.96 | 54.40 | - | - | + | - | CM |
| 7 | F | 54 | - | 4 | Slurred speech | NA | 128 | 3010 | 0.06 | 1.21 | + | + | + | - | CM |
| 8 | M | 40 | - | 60 | Headache | 150 | 151 | 890 | 1.76 | 21.13 | + | + | + | *C.n* | CM |
| 9 | M | 73 | - | 90 | Fever and headache | >330 | 129 | 620 | 0.97 | 16.52 | + | + | + | *C.n* | CM |
| 10 | F | 59 | - | 60 | Fever and headache | >300 | 53 | 600 | 1.36 | 38.53 | + | + | + | *C.n* | CM |
| 11 | M | 39 | - | 14 | Headache | 280 | 133 | 770 | 1.47 | 25.61 | + | - | + | *C.n* | CM |
| 12 | F | 49 | - | 60 | Recurrent headache and vomiting | 190 | 77 | 1090 | 1.35 | 26.52 | - | + | + | *C.n* | CM |
| 13 | F | 72 | - | 10 | Headache and fatigue | 178 | 115 | 650 | 2.47 | 41.24 | + | + | + | - | CM |
| 14 | M | 61 | - | 14 | Headache and fever | 160 | 110 | 2080 | 2.16 | 16.78 | - | - | + | *C.n* | CM |
| 15 | M | 64 | - | 60 | Headache | 130 | 55 | 790 | 1.76 | 35.34 | - | - | + | - | CM |
| 16 | M | 65 | - | 30 | Recurrent fever | 80 | 225 | 930 | 3.92 | 53.70 | + | + | + | *C.n* | CM |
| 17 | M | 27 | - | 10 | Recurrent headache | >330 | 292 | 800 | 2.08 | 30.32 | + | + | + | *C.n* | CM |
| 18 | F | 59 | - | 30 | Recurrent headache and vomiting | 250 | 39 | 640 | 5.64 | 39.52 | + | + | + | - | CM |
| 19 | F | 50 | - | 7 | Recurrent headache | 120 | 186 | 410 | 2.95 | 54.23 | + | + | + | *C.n* | CM |
| 20 | M | 72 | - | 20 | Headache | >330 | 110 | 1000 | 0.02 | 0.30 | + | + | - | - | CM |
| 21 | F | 40 | - | 2 | Abnormal behavior | >300 | 102 | 900 | 0.49 | 8.78 | + | + | + | - | CM |
| 22 | F | 48 | - | 21 | Headache | 30 | 107 | 1800 | 4.94 | 39.43 | + | + | + | *C.n* | CM |
| 23 | F | 70 | - | 60 | Cough and sputum | 210 | 26 | 820 | 5.52 | 42.46 | + | + | + | - | CM |
| 24 | M | 60 | - | 90 | Recurrent headache | 218 | 72 | 950 | 2.46 | 36.18 | + | + | + | - | CM |
| 25 | M | 61 | - | 60 | Headache with fever | 120 | 203 | 1270 | 2.96 | 34.22 | + | + | + | - | CM |
| 26 | F | 71 | - | 30 | Recurrent headache and vomiting | 80 | 274 | 640 | 1.15 | 21.10 | + | + | + | *C.n* | CM |
| 27 | M | 55 | - | 20 | Headache, dizziness and unstable walking | 264 | 52 | 1070 | 2.37 | NA | + | + | + | - | CM |
| 28 | M | 61 | - | 60 | Weakness of limbs | 95 | 171 | NA | 1.69 | NA | + | + | + | *C.n* | CM |

Abbreviation: No., case number; M, male; F, female; HIV, Human Immunodeficiency Virus; SD, symptom duration before admission (days); P, Intracranial pressure; WBC, white blood cell; IIS, India ink stain; Cul, culture; CrAg, cryptococcal antigen; mNGS, metagenomic next-generation sequence; NA, not available; -, negative; +, positive; *C.n*, *Cryptococcus neoformans*; CM, cryptococcal meningitis.
