# Supplementary material for: Single-cell immune landscape of cerebrospinal fluid in non-HIV cryptococcal meningitis
Source: BMC Med. 2026 Aug 10;24:627. doi: 10.1186/s12916-026-05142-x (PMC13746546; doi:10.1186/s12916-026-05142-x)
Supplement: Supplementary file 4 [file 12916_2026_5142_MOESM4_ESM.docx]

**Table S3** Main symptoms and biochemical changes of CSF in pretreated and posttreated CM patients (n = 5).

|  | UCM | TCM | *p** |
| --- | --- | --- | --- |
| Symptoms | | | |
| Fever, n (%) | 5/5 (72.73%) | 0/5 (0%) | NA |
| Headache, n (%) | 5/5 (36.36%) | 0/5 (0%) | NA |
| Altered mental status, n (%) | 2/5 (27.27%) | 0/5 (0%) | NA |
| CSF, median (range) | | | |
| Pressure, mmH_2_O | 158 (80-330) | 176.3 (95-240) | 0.695 |
| White blood count, ×10^6^/L | 154.5 (109-225) | 23 (11-39) | 0.0009 |
| Glucose, mmol/L | 2.44 (0.49-3.92) | 3.09 (2.34-3.45) | 0.155 |
| CSF/blood glucose ratio (%) | 35.97 (8.78-54.22) | 49.91 (36.91-60.43) | 0.037 |
| Proteins, g/L | 1.143 (0.41-2.18) | 0.46 (0.26-4.23) | 0.06 |

Abbreviation: NA, not available; *were compared between UCM and TCM groups using paired t-test.
