# Supplementary material for: Single-cell immune landscape of cerebrospinal fluid in non-HIV cryptococcal meningitis
Source: BMC Med. 2026 Aug 10;24:627. doi: 10.1186/s12916-026-05142-x (PMC13746546; doi:10.1186/s12916-026-05142-x)
Supplement: Supplementary file 5 [file 12916_2026_5142_MOESM5_ESM.docx]

**Table S4** The expression of marker genes for subtypes of cell clusters

| **Cell type** | **Markers** | **Reference (PMID)** |
| --- | --- | --- |
| Bmem Cells | *MS4A1, TNFRSF13B, ANXA2* | 32603654 |
| NaiveB Cells | *IGHD, IGHM, TCL1A* | 31311926 |
| Plasma Cells | *JCHAIN, MZB1, IGHG1* | 33750785 |
| CD4^+^ Temra Cells | *CD4, HOPX, GZMA* | 34914499 |
| CD4^+^ NaiveT Cells | *CCR7, SELL, CD4* | 33771991 |
| CD4^+^ Treg Cells | *FOXP3, IL2RA, CTLA4* | 31937773 |
| CD8^+^ Tem Cells | *GZMK, CD8A, PRF1* | 31118283 |
| γδ T Cells | *TRDC, TRDV2, TRGV9* | 34129837 |
| Proliferating T Cells | *MKI67, TOP2A, STMN1* | 32060101 |
| ILC3 Cells | *IL1R1, KIT, RORC* | 31209406 |
| NK Cells | *NKG7, GNLY, FCGR3A* | 32973147 |
| Macrophages | *CD68, CD163, CD14* | 34031395 |
| Monocytes | *LYZ, FCGR3A, FCN1* | 31937773 |
| MatureDCs | *LAMP3, CCR7, CD83* | 31345789 |
| cDC1 | *CLEC9A, XCR1, IRF8* | 32686767 |
| cDC2 | *CD1C, CLEC10A, FCER1A* | 33782623 |
| pDCs | *CLEC4C, IL3RA, LILRA4* | 32060101 |
