# Supplementary material for: Single-cell immune landscape of cerebrospinal fluid in non-HIV cryptococcal meningitis
Source: BMC Med. 2026 Aug 10;24:627. doi: 10.1186/s12916-026-05142-x (PMC13746546; doi:10.1186/s12916-026-05142-x)
Supplement: Supplementary file 6 [file 12916_2026_5142_MOESM6_ESM.docx]

**Table S5** Genes used for the M2 score

Gene Name

*ARG1*

*ARG2*

*IL10*

*CD32*

*CD163*

*CD23*

*CD200R1*

*PDCD1LG2*

*CD274*

*MARCO*

*CSF1R*

*MRC1*

*IL1RN*

*IL1R2*

*IL4R*

*CCL4*

*CCL13*

*CCL20*

*CCL17*

*CCL18*

*CCL22*

*CCL24*

*LYVE1*

*VEGFA*

*VEGFB*

*VEGFC*

*VEGFD*

*EGF*

*CTSA*

*CTSB*

*CSTC*

*CTSD*

*TGFB1*

*TGFB2*

*TGFB3*

*MMP14*

*MMP19*

*MMP9*

*CLEC7A*

*WNT7B*

*FASL*

*TNFSF12*

*TNFSF8*

*CD276*

*VTCN1*

*MSR1*

*FN1*

*IRF4*
